# P-2136. Bronchoalveolar Lavage *Mucorales* PCR Testing in Immunocompromised Patients with and without Mold Infections

**DOI:** 10.1093/ofid/ofae631.2291

**Published:** 2025-01-29

**Authors:** Ulrike Glatz, Karl Dichtl, Sarah Sedik, Harald Kessler, Matthias Egger, Robert Krause, Martin Hoenigl, Juergen Prattes

**Affiliations:** Medical University of Graz, Graz, Steiermark, Austria; Max von Pettenkofer-Institut für Hygiene und Medizinische Mikrobiologie, Munich, Bayern, Germany; Medical University of Graz, Graz, Steiermark, Austria; Medical University of Graz, Graz, Steiermark, Austria; Medical University of Graz, Graz, Steiermark, Austria; Medical University of Graz, Section of Infectious Diseases and Tropical Medicine, Department of Internal Medicine, Graz, Steiermark, Austria; Division of Infectious Diseases, Department of Internal Medicine, Medical University of Graz, Graz, Austria, Graz, Steiermark, Austria; Medical University of Graz, Graz, Steiermark, Austria

## Abstract

**Background:**

Early detection of mucormycosis is crucial for successfull treatment. Aim of the study was to evaluate the added clinical value of MucorGenius® PCR testing for detection of mucormycosis in bronchoalveolar lavage fluid (BALF) samples from patients at risk for invasive mold infections (IMI).

**Methods:**

A total of 670 BALF samples collected between 2013-2023 from 638 patients were routinely tested for fungal culture and galactomannan before storage at -80°C. IMI was classified according to the FUNDICU 2024 criteria for intensive care unit (ICU) patients (n=277), or the EORTC/MSG 2020 criteria for patients outside the ICU (n=393) .

BALF samples were retrospectively tested with the quantitative real-time MucorGenius® PCR assay between December 2023 and February 2024, and results compared to routine diagnostics and clinical classification

**Results:**

Out of 670 BALF samples, 11 (1.6%) tested positive with the MucorGenius® PCR (mean ct value 32.95), 653 (97.5%) tested negative, while PCR was inhibited in 6 samples (0.9%).

The 11 samples that tested positive included both samples with Mucorales growth in BALF culture. 3/11 samples showed growth of *Aspergillus* spp. (versus 29/653 samples that tested MucorGenius® negative), with BALF GM testing positive ( >0.5 ODI) in 5/5 samples tested, while serum GM was positive in 2/7 samples and *Aspergillus* PCR positive in 1/1 sample. Overall 8/11 samples had mycological evidence for IMI from routine diagnostics.

Of 11 patients with positive MucorGenius® PCR, 6 (55%) met criteria of probable IMI (4 aspergillosis, 2 mucormycosis), while one met criteria of possible IMI. Of those with negative MucorGenius® PCR, 21/653 samples (3.2%) were classified as probable/proven aspergillosis. Therefore 4/25 (16%) of cases classified as probable/proven aspergillosis showed evidence of Mucorales co-infection when MucorGenius® PCR was applied.

**Conclusion:**

Our study outlines the potential value of Mucorales PCR testing in BALF in patients at risk for IMI. The test was highly specific, detected both cases of probable mucormycosis, indicated Mucorales as causative pathogen in a patient with clinical and radiological evidence for IMI but negative routine test results, and detected mucormycosis co-infection in 16% of cases classified as probable/proven aspergillosis.

**Disclosures:**

Martin Hoenigl, MD, Aicuris: Advisor/Consultant|Astra Zeneca: Honoraria|Gilead: Grant/Research Support|Gilead: Honoraria|IMMY: Grant/Research Support|Melinta: Grant/Research Support|Melinta: Honoraria|MSD: Grant/Research Support|Mundipharma: Grant/Research Support|Mundipharma: Honoraria|Pfizer: Grant/Research Support|Pulmocide: Advisor/Consultant|Pulmocide: Grant/Research Support|Scynexis: Advisor/Consultant|Scynexis: Grant/Research Support|Shionogi: Honoraria Juergen Prattes, MD, PhD, AbbVie Inc.: Stocks/Bonds (Private Company)|Gilead: Honoraria|MSD: Grant/Research Support|Novo Nordisk: Stocks/Bonds (Private Company)|Pfizer: Grant/Research Support|Pfizer: Honoraria

